# Towards Effective Patient Simulators

**DOI:** 10.3389/frai.2021.798659

**Published:** 2021-12-15

**Authors:** Vadim Liventsev, Aki Härmä, Milan Petković

**Affiliations:** ^1^ Eindhoven University of Technology, Eindhoven, Netherlands; ^2^ Philips Research (Netherlands), Eindhoven, Netherlands

**Keywords:** reinforcemenet learning, healthcare, simulators and models, clinical methods, markov decision chain

## Abstract

In this paper we give an overview of the field of patient simulators and provide qualitative and quantitative comparison of different modeling and simulation approaches. Simulators can be used to train human caregivers but also to develop and optimize algorithms for clinical decision support applications and test and validate interventions. In this paper we introduce three novel patient simulators with different levels of representational accuracy: HeartPole, a simplistic transparent rule-based system, GraphSim, a graph-based model trained on intensive care data, and Auto-ALS—an adjusted version of an educational software package used for training junior healthcare professionals. We provide a qualitative and quantitative comparison of the previously existing as well as proposed simulators.

## 1 Introduction

Patient simulators have been applied extensively in nursing education (Wendy et al., 2001; Nehring and Lashley, 2004; McFetrich, 2006), both as physical mannequins and as digital patient simulation software (Lee et al., 2020). However, patient simulators have another important application: they can be used to predict how patients’ health would respond to various treatments and thus evaluate proposals for novel treatment strategies. And now, with the advent of artificial intelligence, one can use reinforcement learning (Yu et al., 2019), genetic programming (Koza, 1994; Niazkar and Niazkar, 2020) or both (Liventsev et al., 2021) to generate treatment strategies that lead to the best outcomes for the patient according to a certain simulator. In this paper we review the field of interactive patient simulators and evaluate their potential to be used for automated treatment discovery.

The contributions of this paper are as follows:

• A systematic review of patient simulators in Healthcare Informatics
• *Auto-ALS*—a learning aid for junior healthcare professionals adapted to reinforcement learning
• *HeartPole*—a novel simple and transparent pseudosimulator designed to be a convenient benchmark
• *GraphSim*—a data-driven graph-based simulator based on MIMIC-IV dataset (Johnson et al., 2021) for maximal accuracy

### 1.1 Scope of This Survey

This survey only includes interactive patient simulators where a (human or software) agent communicates its clinical intervention decisions to the simulator and the simulator, in turn,

1) *Predicts* future patient state given current patient state and clinical interventions.
2) *Rewards* or punishes (negatively rewards) that judges if the result of the treatment is positive or negative. The model can be as simple as just negatively rewarding patient deaths or as complex as using the predictions of the *prediction model* to help the agent recognize during the episode whether they are on the right track to a positive outcome.

Any interactive simulator with a prediction model and a reward model (and thus, all simulators in this paper) can be represented as *Episodic Partially Observable Markov Decision Process* (EPOMDP) as described in (Liventsev et al., 2021):

$$\begin{array}{l} M=\left(S_{nt},S_{t},A,O,p_{o}\left(o|s,a\right),p_{s}\left(s_{\text{next}}|s_{\text{prev}},a\right),p_{r}\left(r|s,a\right),p_{\text{init}}\left(s\right)\right) \end{array}$$ (1)

Here, $S_{nt}$ is the set of *non-terminal patient states*. $S_{t}$ is the set of *terminal patient states* (death, discharge from hospital or end of outpatient treatment) such that getting to one of these states signifies the end of an episode. A is the set of *actions* that the learning agent can perform, and O is the set of *observations* about the current state that the agent can make. Distributions *p*_*o*_, *p*_*s*_, and *p*_init_ represent the simulator’s prediction model, while *p*_*r*_ represents the reward model.

Markov Decision Processes are a standard formalism in the field of Reinforcement Learning (Sutton and Barto, 2018), so standard RL algorithms can be used in conjunction with any simulator from this paper for treatment discovery.

There are 2 notable examples of systems that can be referred to as patient simulators, but do not make it easy to evaluate treatment strategies and fall outside the scope of this survey:

1) prediction models without a reward model
2) synthetic data generators (Chen et al., 2021)

They former are tools like HumMod (Hester et al., 2011) that help predict how change in one variable of patient health can affect another, for instance, how digoxin intake affects blood pressure. A reward model is absolutely necessary for the application area that we focus on in this paper—development and validation of treatment strategies. Developing it is also a non-trivial problem in its own right—which health outcomes are considered *good* depends on severity of the patient’s condition (*alive* is a sufficiently successful outcome for the most severe conditions, but not for others) as well as the patient’s own preferences (Street et al., 2012; MühlbacherMühlbacher and Juhnke, 2013). Thus, we view prediction models as components that can be used to develop a full simulator in the future.

The latter simulate clinical scenarios by generating samples of clinical histories. They provide a privacy-preserving way to generate a lot of training data for treatment discovery (James et al., 2018; Gong et al., 2020; Rankin et al., 2020; Wang et al., 2021), however, they don’t model counterfactuals. That is, they don’t answer questions of the sort “How would the patient’s health respond to decision X”? For example, Synthea (Cavalcante et al., 2016) is a framework for generation of entire synthetic Electronic Health Records, EHRs, of patients, from the first to the last patient encounter, but the system does not provide ways to test alternative interventions on patients.

### 1.2 Simulators as Models

What makes an *effective* patient simulator? We argue that there are two use cases for a patient simulator: modeling and benchmarking, with divergent effectiveness criteria.

Firstly, a simulator can be used as a *patient model* aiming to represent the real world evolution of patient health in response to various treatments as closely as possible. The effectiveness criterion for a patient model is simple—*accuracy*. Accuracy can be measured by comparing the simulator’s predictions against a dataset of clinical histories. One metric in particular that has been used for simulator validation is *mean predictive error over dynamic transitions* (Summers et al., 2009). If the simulator was obtained with machine learning, the data also has to be different from the training data: a different dataset or a holdout set can be used for that purpose.

**FIGURE 1:**
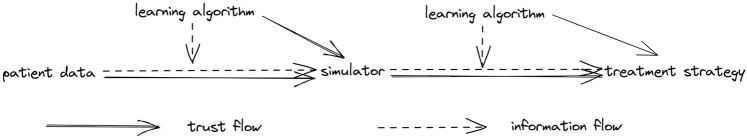
Trust graph of simulator as a model.

The trust graph (see Figure 1) for a model is equally simple: if the data used for development of the simulator is a representative sample of the true distribution of the patients and the methodology used to turn build an interactive simulator based on these data is sound, the simulator is an accurate representation of a real world patient. And if the training algorithm used to discover the optimal treatment under this simulator is sound, the resulting treatment strategy can be trusted as well. Unfortunately, these conditions often hold only partially, or do not hold at all.

### 1.3 Common Biases

One type of bias, present in most data-driven healthcare simulators is *sampling bias*. If patient data is used to develop a simulator, this data is a sample (and, potentially, a biased sample) of the patient population that may or may not represent the population accurately. This problem is exacerbated by a profound shortage of healthcare datasets available to the research community. The biggest currently available dataset is MIMIC-IV (Johnson et al., 2021)—a large database of electronic health records in intensive care. However, MIMIC-IV was collected in one hospital in Boston and thus represents a demographically biased sample of the population. This bias is likely to percolate into any simulator developed based on MIMIC-IV.

But the most crippling type of simulator bias that makes many simulators completely unsuitable to serve as patient models is *confirmation bias*. *Confirmation bias* occurs when the developers of a simulator are aware of state of the art clinical practices and, intentionally or unintentionally, develop a simulator that rewards them and punishes alternatives. In *didactic* simulators used for education of clinical professionals, confirmation bias exists by design: students are, after all, trained to follow established clinical protocols. For a particular example of intentional confirmation bias in simulator design see *Virtu-ALS* (section 2.3). In this simulator, a decision that violates the existing emergence care protocol will be registered as a mistake, explained as such to the student, and *not actually implemented*.

### 1.4 Simulators as Benchmarks

**FIGURE 2:**
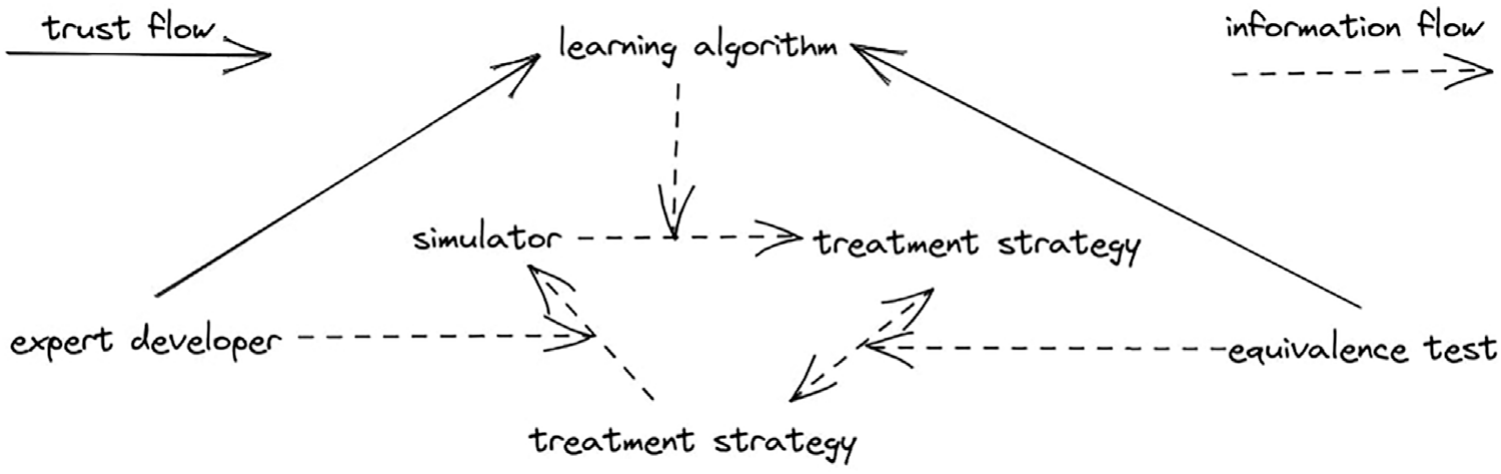
Trust graph of simulator as a benchmark.

Heavily biased and otherwise inaccurate simulators should not be used as patient models and should not be used as a basis for treatment recommendations. However, they can make a great *benchmarks* for learning algorithms. A benchmark is used not to develop a novel treatment strategy and apply it in the real world, but to compare different machine learning algorithms for healthcare against each other. Simulators that exhibit confirmation bias are a good fit for this, since they are similar to true patient models, but unlike in a true patient model, treatments that these simulators evaluate as optimal are known beforehand. One can test a learning algorithm by applying it to a biased simulator and checking that the resulting strategy is equivalent to the strategy that simulator developers had in mind, as displayed on the trust graph in Figure 2.

A crucial metric for effective benchmarks is their *difficulty*. A *difficult* simulator is one where most learning algorithms fail to discover an effective treatment strategy, i.e. a strategy that leads to positive health outcomes. A benchmarks’s purpose is separating stronger learning algorithms from weaker ones, hence a good benchmark has to be not too difficult and not too easy. To get a grasp on simulator difficulty we train a baseline reinforcement learning model on each, see section 4.

Another desideratum for benchmarks is *transparency*. When a learning algorithm fails to discover an effective treatment strategy, it is very useful to understand what went wrong in detail. Any information output by the simulator other than its predictions as well as having easy access to its internal logic can be useful for developers.

Didactic simulators can be particularly good benchmarks (Liventsev, 2021a), but synthetic games akin to CartPole (Barto et al., 1983) and MountainCar (Moore, 1990) (commonly used as benchmarks for Reinforcement Learning algorithms) that aim to mimic only certain aspects of real life healthcare tasks can also be used. A particularly illustrative example is *Healing MNIST* (Krishnan et al., 2015)—a modified version of the industry standard MNIST Handwritten Digits (Li Deng, 2012) dataset to which rotations and random noise have been added. The authors argue that this dataset reflects important properties of healthcare tasks: rotations represent evolution of patient state over time, while the randomised “squares within the sequences are intended to be analogous to seasonal flu or other ailments that a patient could exhibit that are independent of the actions and which last several timesteps.” Due to the data shortage in Healthcare many proposals for data-driven clinical decision support systems are tested on such relatively unrealistic benchmarks with the assumption that the system can be retrained on real patient data and simulators shall they arrive in the future.

## 2 Existing Simulators

### 2.1 Simglucose

**FIGURE 3:**
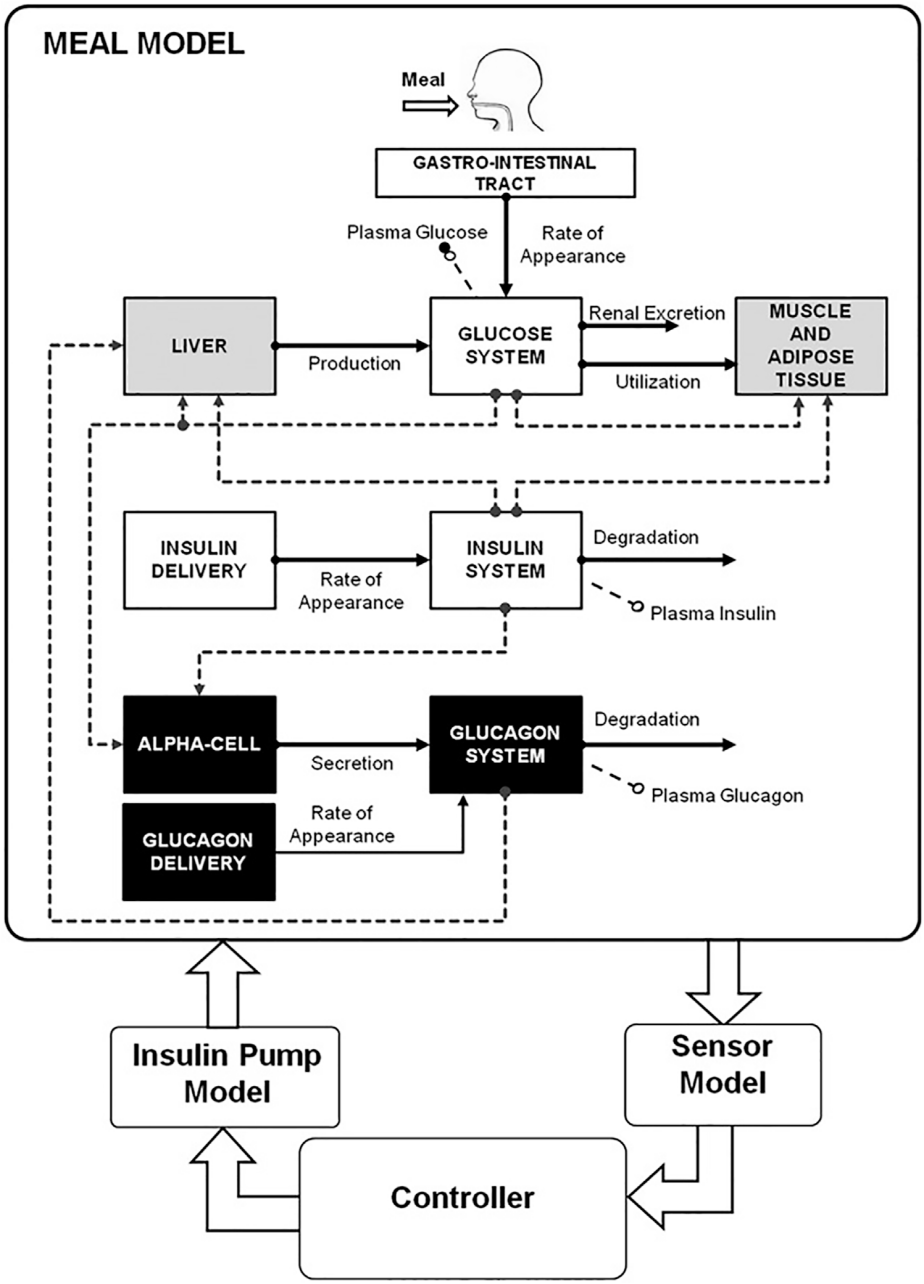
UVA/PADOVA equations, visualised.

UVA/Padova (Man et al., 2014) is a set of equations used to model type 1 diabetes. The equations, outlined on Figure 3, were developed by clinical experts and validated on a dataset of 32 people aged 38 ± 12 years. It is widely used in Healthcare and even approved in the United States as a replacement for clinical trials. It provides *p*_*o*_ (*o*|*s*, *a*) and *p*_*s*_ (*s*_next_|*s*_prev_, *a*) (see section 1.1), so to be a full-fledged Markov Decision Process it only need *p*_*r*_ (*r*|*s*, *a*). (Xie, 2018). solves exactly that by adding a reward function based on diabetes risk index as defined in (Clarke and Kovatchev, 2009) to the UVA/Padova simulator, providing a Reinforcement Learning environment for type 1 diabetes.

### 2.2 GYMIC

#### 2.2.1 Scope

GYMIC (Kiani et al., 2019) is, unlike the previous examples, a fully data-driven simulator. It harnesses a subset of MIMIC (Johnson et al., 2021) dataset to address on one of the most challenging problems in emergency care—sepsis. The authors intentionally limit their scope to just sepsis in order to simplify the modelling task as well as because sepsis prevention has been identified as an area where doctors would particularly benefit from electronic decision support (Raghu et al., 2017; Walonoski et al., 2018).

#### 2.2.2 Prediction Model

The prediction model of GYMIC simulator is defined as a solution to the following autoregression task:

1) A clinical history is a sequence of (*s*, *a*) tuples
2) *a* ∈ 0, …24 is one of 25 possible vasopressor or intravenous fluid interventions—a cartensian product of five types of interventions and five dosage quantiles.
3) *s* ∈ *R* ^46^ is the patient’s state at the moment this intervention was administered.
4) Predict the conditional state distribution *p*_*s*_ (*s*_*t*_, *a*_*t*_|*s*_*t*−1_, *s*_*t*−2_, *…*, *s*_1_)

The dataset of clinical histories is produced by a preprocessing algorithm combining together all clinical records from MIMIC that relate to sepsis patients.

**FIGURE 4:**
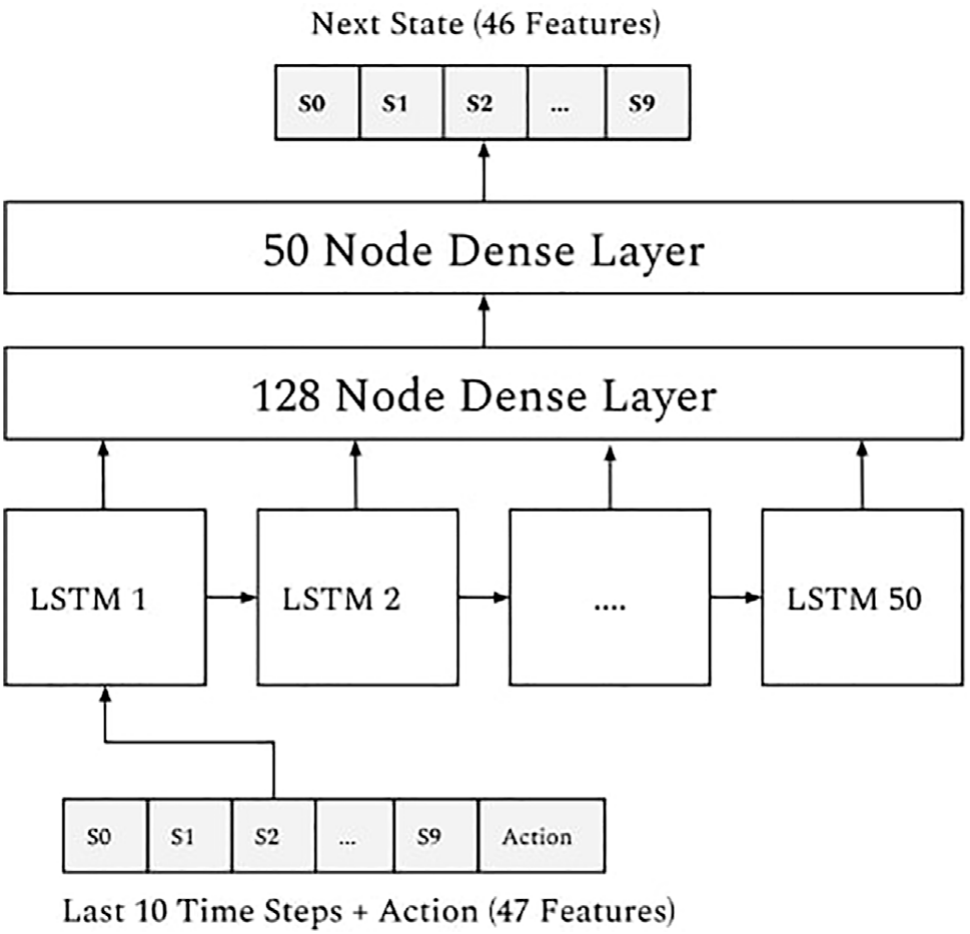
Neural architecture of GYMIC.

Autoregressive tasks of this nature arise in many fields like stock market prediction (Cavalcante et al., 2016; Jiang, 2021) or language modelling (Jozefowicz et al., 2016) where state of the art solutions can be found. The authors of *GYMIC* solve it with an LSTM (Hochreiter and Schmidhuber, 1997) neural network with two additional dense layers attached, see Figure 4 for the diagram. Faced with some of the mode collapse issues described in section 2.2.4 the authors also experimented with semi-supervised learning [? ]: they trained a variational autoencoder [? ] on all patient states to replace the 46-vector representations of patient state *s* with learned representations from the latent space of the VAE encoder(*s*). The issues persisted.

#### 2.2.3 Reward Model

Highlighting the gravity of contracting sepsis, *GYMIC* has only two outcomes: patient is discharged from intensive care or patient dies. Its reward model reflects that, giving the agent a large positive or negative reward at the end of the episode, depending on the outcome. However, in order to lower the difficulty of the simulator (delayed gratification makes training significantly harder (Mischel and EbbesenEbbesen, 1970; Gulwani et al., 2017)) an additional reward is provided during the episode, based on the evolution of the patient’s SOFA score (Lambden et al., 2019)—a commonly used measure of sepsis severity:

$$\begin{array}{l} r\left(s_{t},s_{t+1}\right)=C_{0}1\left(s_{t+1}^{\text{SOFA}}=s_{t}^{\text{SOFA}}\&s_{t+1}^{SOFA}>0\right)+\\ +C_{1}\left(s_{t+1}^{\text{SOFA}}-s_{t}^{\text{SOFA}}\right)+C_{2}\tanh\left(s_{t+1}^{Lactate}-s_{t}^{Lactate}\right) \end{array}$$ (2)

A third reward component is proposed to negatively reinforce action severity and encourage the agent to use low doses of drugs - an instance of *confirmation bias* as discussed in section 1.3, but a necessary step given the issues in section 2.2.4.

#### 2.2.4 Results and Issues

Unfortunately, the experiments performed by the authors of *GYMIC* indicate extreme overfitting. Due to *sampling bias* and simply inadequate size of the dataset there are treatments that have only occurred a few times in the training data and have always resulted in a positive health outcome. In *GYMIC* these treatments are silver bullets that guarantee a successful outcome while in real life they are risky and potentially very harmful.

### 2.3 Virtu-ALS

**FIGURE 5:**
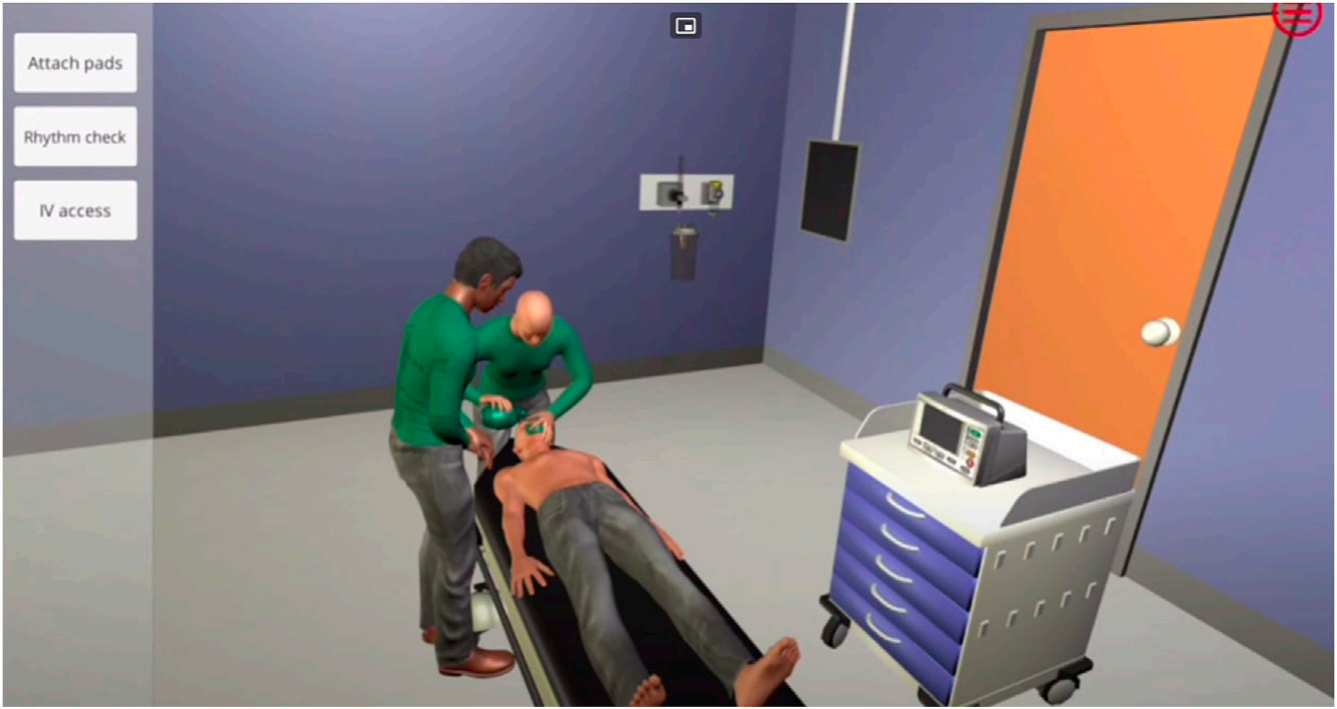
Virtu-ALS.

Virtu-ALS is a *didactic* emergency care simulator mainly targeted at students and junior healthcare professionals, although its application as a reinforcement learning *benchmark* was anticipated and accounted for by the authors (Brisk et al., 2018). Its most prominent feature is its visual nature (Figure 5): the user has access to a 3D-rendered virtual copy of a hospital room, view the monitor, press buttons on a defibrillator, etc. However, the visual modality means that its observation space

$$O\subset R^{307200}$$ (3)

Such a high dimensionality of the observation space makes it an extremely challenging reinforcement learning task. Tasks from this family have been solved with deep neural networks (Mnih et al., 2013), however not only does it require a long and expensive training process, it also means that resulting treatment strategies are black box neural networks that no clinical expert understands. This approach to decision making is extremely hard to introduce into clinical practice (Price, 2018; Watson et al., 2019).

Like most *didactic* simulators, Virtu-ALS exhibits considerable *confirmation bias*—any decision that’s not supported by the standard emergency care protocol (American Heart Association Staff, 2006; Thim et al., 2012) is considered a mistake and rewarded negatively.

## 3 Proposed Simulators

### 3.1 Auto-ALS

As our first model, we propose a low-dimensional version of *Virtu-ALS*. *Auto-ALS* is a modification of Virtu-ALS that removes all the complexity of dealing with a visual 3D environment while retaining all the complexity of dealing with a patient that requires emergency care. This is achieved by attaching an event listener to Virtu-ALS that registers all observable events that can occure in the simulator in response to the user’s actions. The events are listed in Table 1, organized by which agent action can trigger which event. *Tick* is a special event that occurs every time the simulator is advanced a timestep, and is negatively reinforced, which when used with reinforcement learning algorithms discourages clinicaly unnecessary actions.

**TABLE 1:** All actions and observations of Auto-ALS.

Agent actions	Patient reactions	Rewards
AssessResponse	ResponseVerbal, ResponseGroan, ResponseNone	0
AssessAirway	AirwayClear, AirwayVomit, AirwayBlood, AirwayTongue	
AssessBreathing	BreathingNone, BreathingSnoring, BreathingSeeSaw, BreathingEqualChestExpansion, BreathingBibasalCrepitations, BreathingWheeze, BreathingCoarseCrepitationsAtBase, BreathingPneumothoraxSymptoms, VentilationResistance, *MeasuredRespRate*	
AssessCirculation	RadialPulsePalpable, RadialPulseNonPalpable, *MeasuredHeartRate*	
AssessDisability	AVPU_A, AVPU_U, AVPU_V, PupilsPinpoint, PupilsNormal, *MeasuredCapillaryGlucose*	
AssessExposure	ExposureRash, ExposurePeripherallyShutdown, ExposureStainedUnderwear, *MeasuredTemperature*	
AssessDefibrillator		
AssessMonitor	HeartRhythm0, HeartRhythm1, HeartRhythm2, HeartRhythm3, HeartRhythm4, *MeasuredHeartRate*, *MeasuredMAP*, *MeasuredSats*, *MeasuredResps*	
DoNothing		
ABG, AirwayManoeuvres, GiveAtropine, GiveAdenosine, GiveAdrenaline, GiveAmiodarone, GiveMidazolam, Venflon, Yankeur, DrawBloods, BPCuffOn, BVM, Guedel, NRBMask, DefibOn, DefibAttachPads, DefibShock, DefibCharge, DefibChangePaceCurrentDown, DefibChangePaceCurrent, DefibEnergyDown, DefibEnergyUp, DefibChangePaceRateDown, DefibChangePaceRateUp, DefibPace	Blunder	*r*_blunder_
Finish	Failure	−1
	Success	1
—	Tick	*r*_tick_

MeasuredHeartRate, MeasuredRespRate, MeasuredCapillaryGlucose, MeasuredTemperature, MeasuredMAP, MeasuredSats, and MeasuredResps are *measurements*, events that have a value (− *∞*; + *∞*) associated with them.

The events in Table 1 only get registered if the agent has *learnt* some piece of information, meaning that, for example, AirwayVomit will only occur if the patient has vomit in their airway *and* the agent checked the airway (which is part of the standard protocol (Thim et al., 2012)). Assessment skills (knowing where to look and how to establish the patient’s state) are crucial for patient resuscitation, hence revealing all known health variables to the agent would jeopardize the simulation.

The observation vector in *Auto-ALS* is based on all observations that have occurred between the beginning of the episode and current time. However, more recent observations are more likely to still be relevant and should be given priority. This is done with the following formula proposed in (Liventsev, 2021b):

$$o^{+}=\left\langle o_{1}\in O_{1},\exp\left(t_{1}-t\right),\ldots ,o_{n}\in O_{n},\exp\left(t_{n}-t\right),\right\rangle$$ (4)

where *O*_*i*_ is the value of the observation and *t* is current time and *t*_*i*_ is time when observation *i* (for *i* = 5, ResponseGroan) has *last* occurred and exp (*t*_*i*_ − *t*) represents its decaying relevance. For *measurements*, the *O*_*i*_ equals the magnitude of the measurement, however, for binary obsevations *O*_*i*_ would always be equal to one. For memory efficiency, for all *i* that correspond to binary observations, *O*_*i*_ is skipped from the *o* ^+^ vector and the actual observation vector *o* has size 36 + 7∗2 = 50, as opposed to (36 + 7)∗2 = 86.

See source code and documentation at (Liventsev, 2021c).

### 3.2 HeartPole

HeartPole focuses on simplicity and transparency at the expense of realism—it is based on a familiar scenario and a simple set of rules so that when a treatment performs badly it is easy to explore what exactly goes wrong. *HeartPole* simulates a creative professional trying to become more productive. However, many decisions that would help in the short term (not sleeping, consuming coffee and alcohol) can create long-term health issues that negate all short term gains.

In *HeartPole* state *s* consists of alertness $s_{t}^{\text{alert}}$ , hypertension *s* ^hypert^, intoxication *s* ^tox^ time since slept *s* ^tawake^, *total time elapsed* *s* ^ttotal^ and *total work done* *s* ^done^.

**FIGURE 6:**
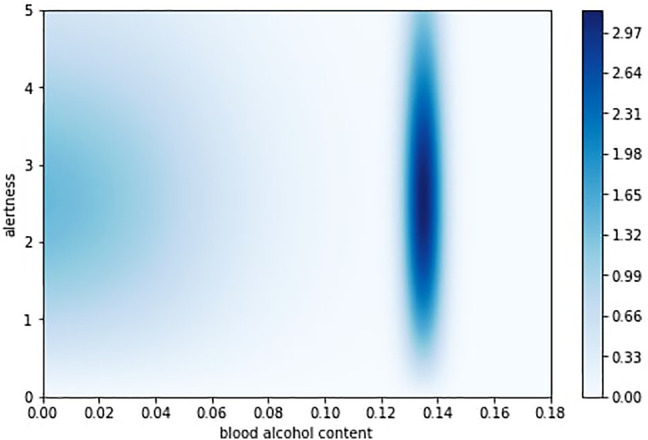
Productivity function in HeartPole.

Over these parameters, we define *productivity* function $\eta \left(s_{t}^{\text{alert}},s_{t}^{\text{tox}}\right)$ presented graphically on Figure 6 and *heart attack probability* $r\left(s_{t}^{\text{hypert}}\right)=\frac{sigmoid\left(s_{t}^{\text{hypert}}\right)}{2}$ . The agent receives small positive rewards for productivity and a very large negative reward if a heart attack occurs.

**FIGURE 7:**
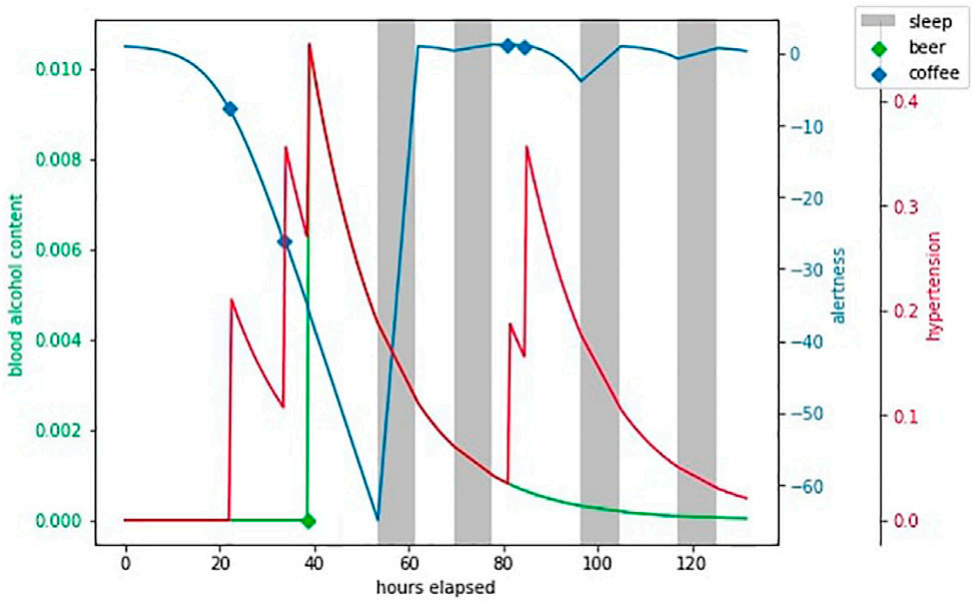
An example episode in HeartPole.

As shown on Figure 7, every half an hour awake, the agent observes *s*_*t*_ and picks an action *a*_*t*_ from discrete action space of *just work*, *drink coffee* (increases *s* ^alert^ and *s* ^hypert^), *drink beer* (decreases *s* ^alert^, increases *s* ^hypert^ and $s_{t}^{\text{tox}}$ ) and *go to bed* (sleep takes a lot of time, but reduces *s* ^hypert^ and $s_{t}^{\text{tox}}$ and without it alertness starts to fall very fast).

See source code and documentation at (Härmä et al., 2021).

### 3.3 GraphSim

Our last model is trained on MIMIC (Johnson et al., 2021) to maximise *accuracy*. MIMIC can be represented as a set of patients where every patient is an oriented graph, its nodes are patient states, each state is a vector of various clinical measurements, such as blood pressure and oxygen saturation, whereas arcs that connect the patient states are doctors actions, each action a vector of administered drug doses. These oriented graphs can also be viewed as disjoint clusters in one graph of all possible patient states, a sequence of patient states connected by doctor agents.

$$G=\left\{\left\langle s_{\text{before}},a,s_{\text{after}}\right\rangle \right\}$$ (5)

Some states in this oriented graph are very similar, it is reasonable to assume that two different patients have been in the same state at some point. Our algorithm is based on the idea that if two patients have been in the same state, their clinical histories represent two possible timelines of events after the state and the choice of timeline depends on doctor’s actions.

**FIGURE 8:**
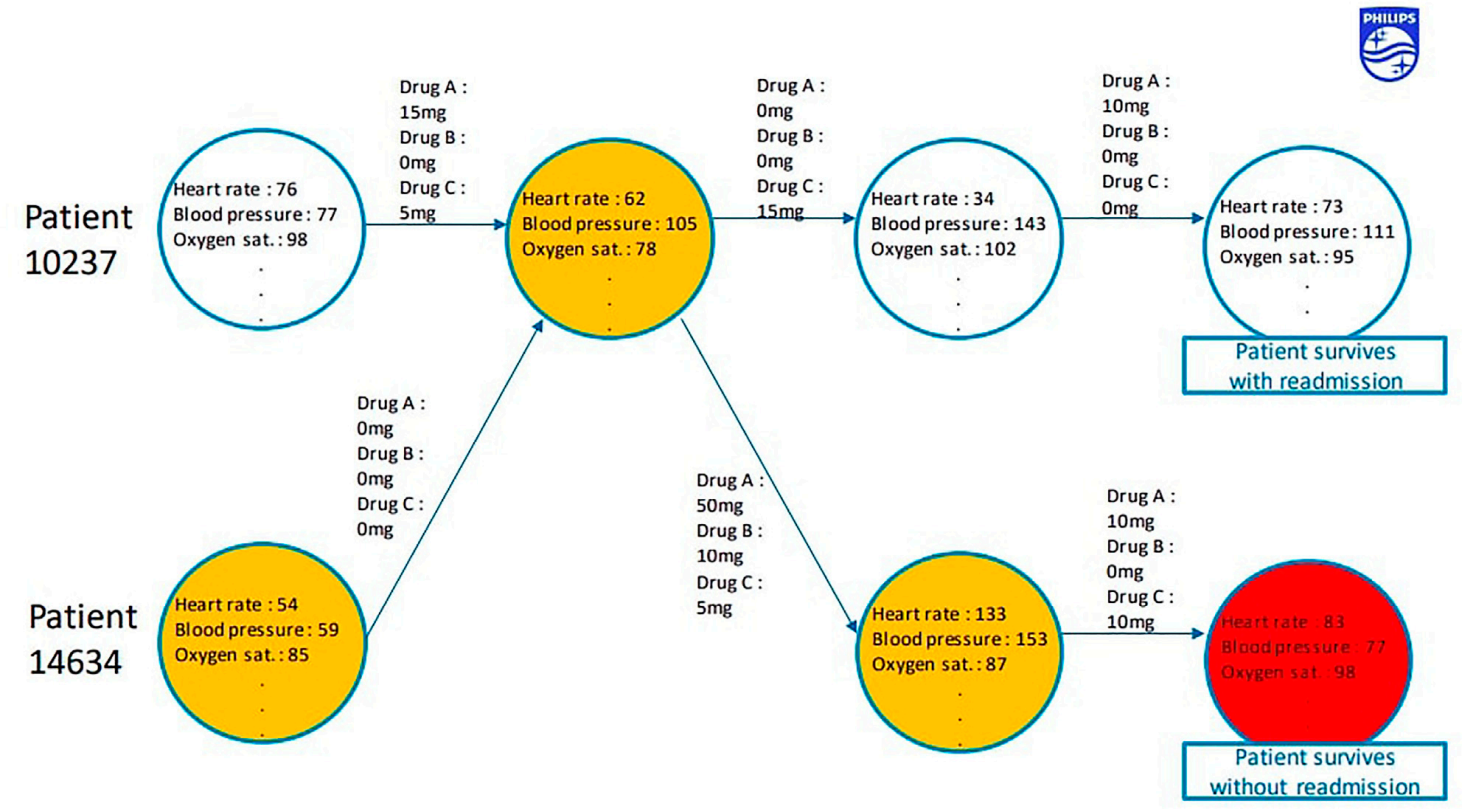
GraphSim.

We find all state pairs ⟨*s*_*A*_, *s*_*B*_⟩ below a similarity threshold

$$\cos\left(s_{A},s_{B}\right)<c_{\text{min}}$$ (6)

and merge each into a single state, replacing occurences of *s*_*A*_ and *s*_*B*_ in *G* with $\frac{s_{A}+s_{B}}{2}$ . The resulting oriented graph becomes the backbone of out simulator. The simulated patient is initialized in state *s*_0_ equal to one of the initial states of real patients in MIMIC. When at timestep *t* the agent picks action *a*_*t*_, transition to the next state depends on euclidean distance between the action in the graph and *a*_*t*_ via the softmax function:

$$p\left(s_{t+1}|s_{t},a_{t}\right)=\frac{\sum_{\langle s_{\text{before}},a,s_{\text{after}}\rangle }^{G}I\left[s_{\text{before}}=s_{t}\right]I\left[s_{\text{after}}=s_{t+1}\right]e^{|a_{t}-a{|}_{2}^{2}}}{\sum_{\langle s_{\text{before}},a,s_{\text{after}}\rangle }^{G}I\left[s_{\text{before}}=s_{t}\right]e^{|a_{t}-a{|}_{2}^{2}}}$$ (7)

where I is the indicator function. See Figure 8 for a diagram of the resulting simulator.

The simulator’s source code will be published soon.

## 4 Effectiveness

How do these simulators fare with respect to *accuracy* criteria we set out in the introduction? The factors that contribute to a simulator’s accuracy are reviewed in Table 2. *GYMIC* and *GraphSim* are the only simulators trained on a large dataset and GYMIC’s accurracy has known overfitting issues. *GraphSim* is thus the most accurate of the simulators.

**TABLE 2:** Summary of each simulator: trust view.

Simulator	Scope	Data source	Sample size	Learning algorithm	Known biases
simglucose (Xie, 2018)	type 1 diabetes	original study	32	expert model validated on data behavior cloning	Overfitting confirmation bias confirmation bias no factual basis
GYMIC (Kiani et al., 2019)	sepsis in intensive care	MIMIC (Johnson et al., 2021)	40 ,000		
Virtu-ALS (Brisk et al., 2018)	emergency care				
Auto-ALS	emergency care				
HeartPole	healthy lifestyle				
GraphSim	intensive care	MIMIC (Johnson et al., 2021)	40 ,000	graph compression	

The most *transparent* simulator is clearly *HeartPole*. It does not aim to model any real clinical scenario accurately, but it can be a useful development tool to help scrutinize reinforcement learning algorithms.

As far as *difficulty* is concerned, *HeartPole* (Härmä et al., 2021), *simglucose* (Zhu et al., 2021), and *GYMIC* (Kiani et al., 2019) are known to be solvable with relatively small models and standard reinforcement learning algorithms like DQN (Mnih et al., 2015). Thus, the only simulators *difficult* enough to be benchmarks for novel approaches are *Virtu-ALS* and *Auto-ALS* and *Auto-ALS* is the more accessible of the two.

Table 3 reviews the structural complexity of the simulators, a factor that directly contributes to *difficulty*. Note that *Virtu-ALS* is an unusually high-dimensional environment. As such, solving it is likely to require more parameters and longer training times. *GraphSim* is the only simulator that doesn’t provide non-zero rewards in non-terminal states *S*_*nt*_, making it harder for the agent to attribute the results of the episode to particular actions. *GYMIC* (see section 2.2) solves this problem with an additional metric (SOFA score), but unlike *GYMIC GraphSim* covers a wide range of clinical conditions and there is no single health metric applicable to each.

**TABLE 3:** Summary of each simulator: POMDP view.

Simulator	O	A	*p*_*r*_ (*r* = 0|*s*) ≠ 1
simglucose	$\left[\left.0;+\infty \right)\right.$	[0; 35]	*S*_*nt*_ ∪ *S*_*t*_
GYMIC	[0; 24]^46^	0, *…* , 24	*S*_*nt*_ ∪ *S*_*t*_
Virtu-ALS	[0; 256]^*307*^ ^*200*^	1, *…* , 307 200	*S*_*nt*_ ∪ *S*_*t*_
Auto-ALS	${\left[\left.0;+\infty \right)\right.}^{36}$	1, *…* , 34	*S*_*nt*_ ∪ *S*_*t*_
HeartPole	$R^{6}$	1, *…* , 4	*S*_*nt*_ ∪ *S*_*t*_
GraphSim	(−*∞*;+*∞*)^26^	[0; 1]^*317*^	*S*_*t*_

## 5 Conclusion

Automatic discovery of clinical strategies is a nascent field of research that has a potential to considerably improve patient outcomes and become a new modus operandi in healthcare research. The goal of this paper is to provide a solid foundation for further development of this field with better patient simulators and better understanding thereof. We have reviewed the state of the art in patient simulators, identified some of the problems the field is facing and proposed novel simulators to address them. We believe that *HeartPole* and *Auto-ALS* can become new standard benchmarks for reinforcement learning in healthcare, while *GraphSim* can become a stepping stone to improved patient outcomes in intensive care.

## Data Availability

Publicly available datasets were analyzed in this study. This data can be found here: https://physionet.org/content/mimiciv/0.4/
